# Immunohistochemical expression of PD1, LAG3, and CTLA4 in diffuse large B cell lymphoma, clinicopathological correlation, and prognostic value

**DOI:** 10.1186/s43046-025-00303-0

**Published:** 2025-06-07

**Authors:** Madonna I. William, Dina A Tantawy, Alyaa R. Elsergany, Amira K El-Hawary, Shaimaa M Yussif

**Affiliations:** Mansoura University, Al Mansurah, Egypt

**Keywords:** DLBCL, Survival, PD1, CTLA4, LAG3

## Abstract

**Background:**

The tumor microenvironment has an important role in the growth and progression of diffuse large B-cell lymphoma (DLBCL). Immune checkpoint molecules, including PD1, LAG3, and CTLA4, are crucial to regulate the T cells function in the tumor microenvironment. Exploring the expression of these molecules in DLBCL microenvironment is crucial for developing targeted therapies enhancing anti-tumor immune responses.

**Aim:**

This study aims to evaluate the immunohistochemical (IHC) expression of PD1, LAG3, and CTLA4 in DLBCL, assess the relation of their expression to different clinicopathological parameters and evaluate their prognostic significance.

**Methods:**

This retrospective study encompassed 103 cases diagnosed as de novo DLBCL. Clinicopathologic and survival data were gathered. IHC for PD1, LAG3, and CTLA4 was performed.

**Results:**

PD1, LAG3, and CTLA4 positive reaction was observed in tumor-infiltrating lymphocytes (TILs) in 68.9% (71/103), 82.5% (85/103), and 92.2% (95/103) of DLBCL cases, respectively. PD1 expression in TILs was significantly associated with hepatitis C virus (HCV) positivity and prolonged overall survival (OS) in univariate analysis. LAG3 expression in TILs was significantly associated with IPI score and tended towards shorter OS (not statistically significant). LAG3 expression in tumor cells was significantly associated with shorter disease-free survival (DFS). CTLA4 expression in TILs was significantly associated with advanced disease stage (III/IV).

**Conclusion:**

PD1 and LAG3 are expressed mainly in TILs. PD1 expression (in TILs and tumor cells) is associated with prolonged OS, while LAG3 expression (in tumor cells) is associated with shorter DFS and its expression in TILs tended towards shorter OS. CTLA4 expression is associated with advanced disease stage but not associated with OS. These findings may suggest that immune checkpoint inhibitors targeting LAG3 may offer therapeutic potential in DLBCL by enhancing the antitumor immune response. Additional research is needed to assess the effectiveness of inhibition of these checkpoint molecules in combination with existing treatment modalities.

## Introduction

Diffuse large B-cell lymphoma (DLBCL) is the most prevalent histologic subtype of non-Hodgkin lymphoma (NHL), accounting for around 30% of NHL cases [1]. In comparison to other B-cell NHL subtypes, DLBCL is more common in Africa than in the developed world [2]. According to the Egyptian National Cancer Institute (NCI), 49% of all cases of NHL in Egypt are DLBCL [3]. The majority of DLBCLs are nodal lymphomas; however, 40% of cases are thought to be primarily extra nodal in origin, with the gastrointestinal tract being the most frequent extra nodal location [4].

The tumor microenvironment (TME) is a highly organized eco-system that is composed of cancer cells encircled by various non-cancerous cell types. These cells are all immersed in a vascularized extracellular matrix that has been altered [5]. The possible significance of TME in DLBCL pathogenesis has been the subject of several recent researches; however, the findings have not been widely accepted [6]. In addition to traditional risk categorization criteria like the international prognostic index (IPI), cell of origin, and molecular changes, TME composition has a significant influence on the onset, progression, and expansion of DLBCL [7].

In the tumor microenvironment, immune checkpoints (IC) are vital molecules that control T cell function. Immune checkpoint therapy has significantly changed the way that cancer is treated by blocking T cell inhibitory mechanisms to enhance anti-tumor immune responses [8].

Programmed cell-death protein 1 (PD1), cytotoxic T-lymphocyte-associated protein 4 (CTLA4) and, lymphocyte-activation gene-3 (LAG3) are members of IC molecules that are recognized as essential modulators of the immune response [9]. PD1, expressed on the surface of activated T-cells via T cell receptor downstream signaling, plays a vital role in controlling T-cell-directed anti-tumor immune responses in TME. It binds the ligand PD-L1 and then sends inhibitory signals to control T-cell activation [10].

Constitutively expressed in T regulatory cells (Tregs), CTLA4 is a crucial negative regulator of T-cell activation. It promotes tumor cell survival and suppresses the proper immune response in the TME [11].

Major histocompatibility complex class II (MHC-II) molecules, which are present on the surface of tumor cells and antigen-presenting cells, are the primary ligands of LAG3 [12]. When LAG3 and its ligand interact, the antigen-specific CD4 + T-cell response is down regulated, and cytokine production is decreased, which adversely affects T cell activation and proliferation [11].

The therapeutic role of IC inhibition in DLBCL is currently unclear and necessitates further research. The purpose of our study is to evaluate the immunohistochemical (IHC) expression of PD1, LAG3, and CTLA4 in DLBCL and its correlation with clinicopathological parameters and survival outcomes.

## Material and methods

This retrospective study was conducted on 103 cases diagnosed as de novo DLBCL, NOS (not otherwise specified) during the period from 2013 to 2022 at the pathology laboratory, Oncology Centre of Mansoura University (OCMU), Egypt. Cases with available clinical data and paraffin blocks, diagnosed by excised lymph node biopsy were involved in this study. Extra-nodal cases and cases diagnosed by tru-cut biopsy or fine needle aspiration cytology were excluded. Follow up period extended up to 2024.

Hematoxylin and eosin (H&E) and IHC stained slides (CD20 and CD3) were obtained from the slides’ archive then examined for diagnosis confirmation.

Slides stained for CD10, BCL6, and MUM-1 were examined to assess the cell of origin and classify the cases to germinal center origin and non-germinal center origin based on Hans’ algorithm [13].

The clinical and laboratory findings of the selected cases were collected from the pathologic and clinical databases of OCMU. The clinical data included age, gender, presence of comorbidity, serum lactate dehydrogenase (LDH) level, B symptoms, Ann Arbor stage, molecular type, the number of extranodal sites involved, international prognostic index (IPI), bone marrow aspirate and biopsy involvement, and treatment modality.

The survival data included the overall survival (OS) defined as the time interval from the date of diagnosis to the patient’s last follow-up appointment or the time of disease-specific death. The disease-free survival (DFS) was defined as the time interval between the diagnostic date and the date of progression or relapse. The follow-up period extended up to 2024.

### Tissue microarray (TMA) construction

To choose the most typical regions, the histologic characteristics of the entire mount H&E slides were examined. Using a permanent marker, selected regions were ringed on the H&E slides. The same regions were subsequently identified on the matching paraffin blocks.

Three TMA blocks were assembled manually [14], each one including the 103 selected cases of DLBCL. As orientation and navigation markers, several cores of normal tissues—the kidney, lymph node, appendix, placenta, and brain—were placed in accordance with a pre-made map. Tonsils tissue cores were placed in every block to be used as positive and negative controls for PD1 and LAG3 [15] and CTLA4 [16].

#### Immunohistochemistry:

TMA Sects. (4 μm) were immunostained with the DAKO autostainer using the following antibodies:

- Anti-PD1 antibody: rabbit monoclonal antibody (Quartett, Berlin, Germany, 1:100, clone QR2, ready to use).
- Anti-LAG3 antibody: rabbit polyclonal antibody (Quartett, Posydam, Germany, 1:100, ready to use).
- Anti-CTLA4 antibody (F8): mouse monoclonal antibody (Medaysis, San Francisco, USA, 1:100, ready to use).

IHC was carried out with the Autostainer Link 48, using its optimized reagents and the pharmDx kits EnVision™ FLEX Visualization Systems (Link code K8000) and EnVision FLEX Hematoxylin (Link code K8008), following the standardized procedure programmed into the autostainer software as per the user’s guide.

#### IHC interpretation

Evaluation of PD1, LAG3, and CTLA4 expression in tumor-infiltrating lymphocytes (TILs) and tumor cells was performed.

##### Tumor-infiltrating lymphocytes

Tumor-infiltrating lymphocytes expressing membranous or cytoplasmic positivity for PD1, LAG3 and cytoplasmic positivity for CTLA4 were counted in three high power fields (in areas of highest density) and the average number was calculated [15] [17].

##### Tumor cells

Tumor cells expressing cytoplasmic or membranous positivity for PD1 and LAG3, with a cut off 10% was considered positive [15]. In CTLA4, cytoplasmic positivity in ≥ 10% of tumor cells was considered positive [18].

#### Statistical analysis

To analyze the data, IBM SPSS Statistics for Windows, Version 25.0 Armonk, NY: IBM Corp. was used. Numbers and percentages were used to express qualitative data. The chi-square test (*χ*2), Fisher’s exact test, and Monte Carlo test were used to compare between groups, as appropriate. Quantitative data were expressed as mean (SD) or median (minimum-maximum), as appropriate. They were tested for normality using the Kolmogorov–Smirnov test. To estimate the survival function from lifetime data, survival analysis (Kaplan–Meier estimator) is utilized. It is utilized to measure the fraction of patients living for a certain amount of time post treatment and the log rank test was conducted to statistically compare two groups. By calculating the hazard ratio, variables influencing OS and DFS are determined using Cox regression analysis for multivariate analysis. “*P* value < 0.05’’ was interpreted as statistically significant.

### Ethical considerations

The investigation was conducted using archive material from paraffin tissue blocks that were kept in the pathology laboratory. The Institutional Research Board (IRB), code number: MDP.21.10.83 at Mansoura University’s Faculty of Medicine has approved the task proposal. Patient confidentiality was maintained throughout the trial by using their code numbers instead of their names. Lastly, the donor paraffin blocks were restored in the archives for future patient or research use.

## Results

Table 1 summarizes the demographic and clinico-pathological data of the 103 DLBCL patients studied (mean age 53.6 ± 11.2 years, range 12–76; 56 males, 47 females).

**Table 1:** Demographic and clinico-pathological data of the studied cases (*n* = 103)

Clinicopathological parameter		Study group (*n* = 103)
Age (years)	< 60 years ≥ 60 years Mean ± SD (min–max)	70 (68%) 33 (32%) 53.6 ± 11.2 *(12–76)*
Gender	Male Female	56 (54.4%) 47 (45.6%)
Diabetes mellitus	No Yes	95 (92.2%) 8 (7.8%)
HCV	No Yes	62 (60.2%) 41 (39.8%)
B-Symptoms	No Yes	76 (73.8%) 27 (26.2%)
Molecular type	Germinal center Non-germinal center	44 (42.7%) 59 (57.3%)
LDH level	Low (< 190 U/L) High (≥ 190 U/L)	12 (11.7%) 91 (88.3%)
Ann Arbor stage	I II III IV	6 (5.8%) 28 (27.2%) 49 (47.6%) 20 (19.4%)
Extra nodal sites	No One site More than 1 site	77 (74.8%) 22 (21.4%) 4 (3.9%)
IPI score of lymphoma	Low Low-intermediate High-intermediate High	33 (32%) 37 (35.9%) 25 (24.3%) 8 (7.8%)
Bone marrow aspiration (BMA)	No Yes	91 (88.3%) 12 (11.7%)
Bone marrow biopsy (BMB)	No Yes	89 (86.4%) 14 (13.6%)
First line treatment	CHOP R-CHOP	86 (83.5%) 17 (16.5%)
Survival	Alive Dead	22 (21.4%) 81 (78.6%)
Overall survival (OS) time (months)	Median (min–max)	24 (1–132)
Relapse/progression	No relapse Relapse	49 (47.6%) 54 (52.4%)
Disease-free survival (DFS) (months)	Median (min–max)	14 (1–84)

Data expressed as number (%) or mean ± SD

**Fig. 1:**
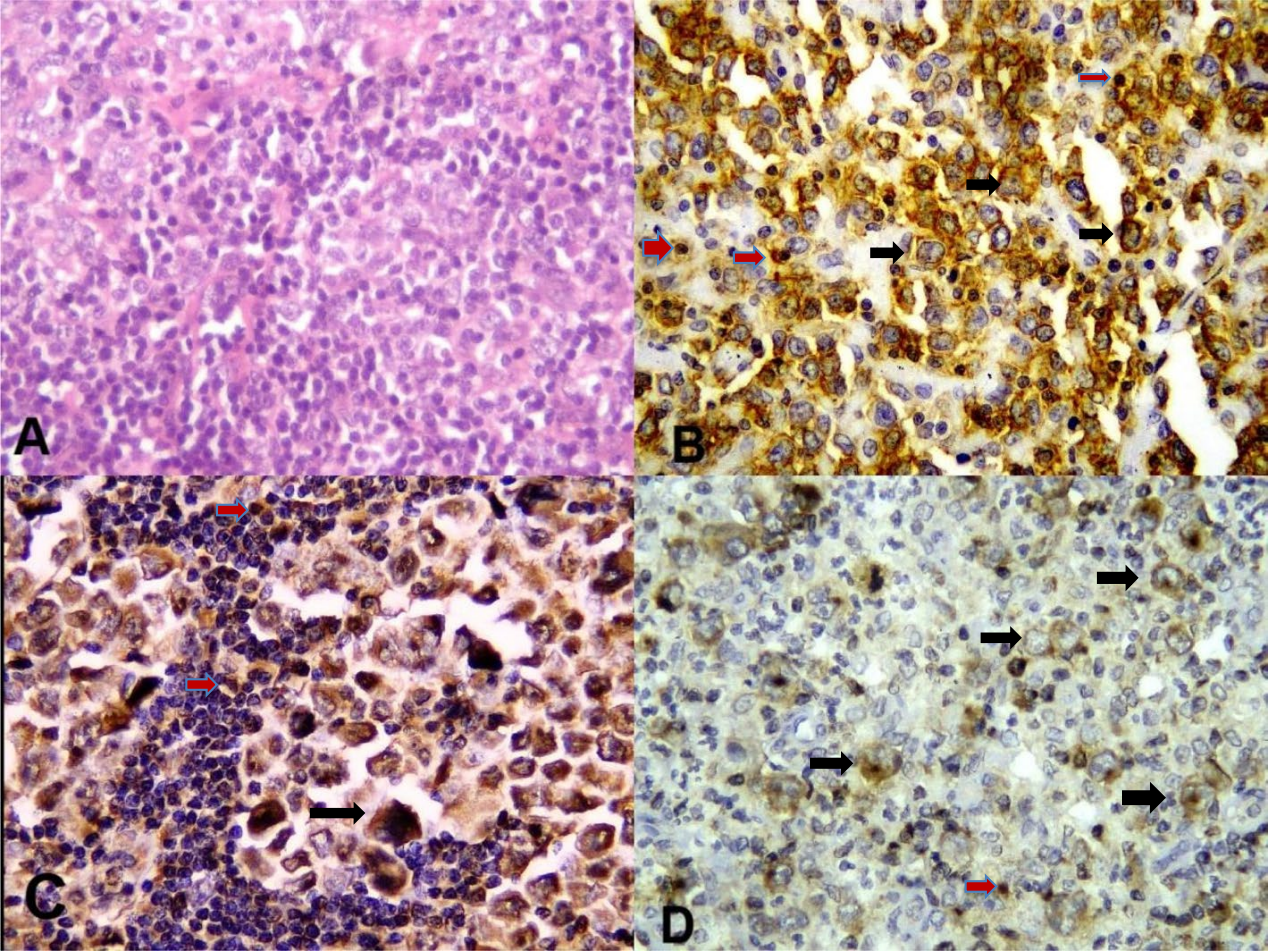
**A** A case of Diffuse large B cell lymphoma showing classical morphology (H&E, × 400), **B** PD1 positive membranous reaction in tumor cells (black arrows) and tumor infiltrating lymphocytes (red arrows) (IHC, × 400). **C** CTLA4 positive cytoplasmic reaction in tumor cells (black arrows) and scattered positive TILs (red arrows) (IHC, × 400). **D** LAG3 positive cytoplasmic reaction in tumor cells (black arrows) and focal positive in TILs (red arrows) (IHC, × 400)

**Fig. 2:**
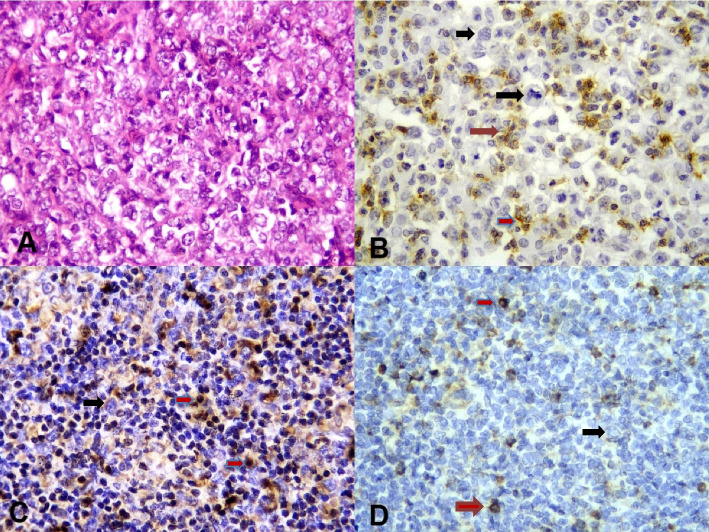
**A** a case of DLBCL showing tumor cells and tumor infiltrating lymphocytes (H&E, × 400). **B** PD1 positive expression in tumor infiltrating lymphocytes (red arrows) and negative expression in tumor cells (black arrows) (IHC, × 400). **C** CTLA4 expression in TILs (red arrows) and negative expression in tumor cells (black arrows) (IHC, × 400). **D** LAG3 expression in scattered TILs (red arrows) and negative expression in tumor cells (black arrows) (IHC, × 400)

PD1 expression in TILs was seen in 71 cases (68.9%) with a median of 10 (range 1–40) cells. Most cases (98/103) (95.1%) were negative for PD1 in tumor cells. LAG3 expression in TILs was positive in 85 cases (82.5%), with a median of 10 (range 1–40) cells; while most cases (99/103) (96.1%) were negative in tumor cells. CTLA4 expression in TILs was detected in 95 (92.2%) cases, with a median of 8 (range 1–50) cells. CTLA4 was positive in tumor cells in 24 cases (23.3%) (Table 2) (Figs. 1 and 2).

**Table 2:** Immunohistochemical expression of PD1, LAG3, and CTLA4 in tumor cells and in tumor infiltrating lymphocytes (TILS) in patients with diffuse large B cell lymphoma (*n* = 103)

Immunohistochemical expression	Study group (*n* = 103) *N* (%)
PD1 expression	
In TILs	
• Negative	32 (31.1%)
• Positive	71 (68.9%)
In tumor cells	
• Negative	98 (95.1%)
• Positive	5 (4.9%)
Value of positive PD1 in TILs	
Median (min–max)	10 (1–40)
LAG3 expression	
In TILs	
• Negative	18 (17.5%)
• Positive	85 (82.5%)
In tumor cells	
• Negative	99 (96.1%)
• Positive	4 (3.9%)
Value of positive LAG3 in TILs	
Median (min–max)	10 (1–40)
CTLA4 expression	
In TILs	
• Negative	8 (7.8%)
• Positive	95 (92.2%)
In tumor cells	
• Negative	79 (76.7%)
• Positive	24 (23.3%)
Value of positive CTLA4 in TILs	
Median (min–max)	8 (1–50)

Parameters of studied cases (*n* = 103)

Positive PD1 reaction in TILs was significantly associated with hepatitis C virus (HCV) positive status (*P* value = 0.003), with 85.4% of HCV-positive patients exhibiting PD1-positive TILs. PD1 expression in TILs and the molecular type of the patients under study were statistically associated (*P* value = 0.04).

Positive LAG3 reaction in TILs was significantly associated with IPI score (*P* value = 0.001), with 96% of high-intermediate risk cases expressing LAG3. A significant association was also noticed between CTLA4 and LAG3 expression in TILs (*P* = 0.03), with 85.3% of CTLA4 positive cases also expressing LAG3.

CTLA4 positive reaction in TILs was significantly associated with disease stage (*p* = 0.04), with all stage IV cases expressing CTLA4 and advanced stages (III/IV) were associated with CTLA4 positivity. While a strong association was observed between CTLA4 expression and HCV positivity (85.4% of HCV positive cases were CTLA4 positive), this association did not attain statistical significance (*P* value = 0.06).

### Survival analysis

**Fig. 3:**
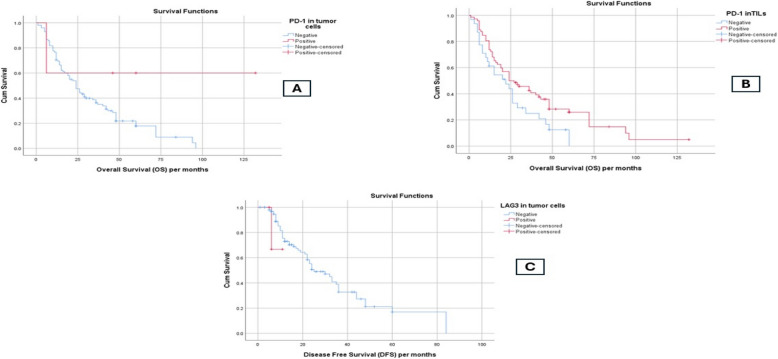
**A** Kaplan-Meir survival curve for cases with diffuse large B cell lymphoma stratified by expression of PD1 in tumor cells, cases with negative PD1 in tumor cells were associated with lower overall survival (OS) (log rank; *P* = 0.03). **B** Kaplan-Meir survival curve for studied cases stratified by expression of PD1 in TILS, cases with negative PD1 in TILS were associated with lower OS (log rank; *P* = 0.04). **C** Kaplan-Meir survival curve for studied cases by LAG3 expression in tumor cells, cases with positive LAG3 were associated with lower disease-free survival (DFS) (log rank; *P* = 0.04)

Univariate analysis showed that diabetes mellitus (median OS 15.3 months, *P* = 0.03) and high IPI score (median OS 13.3 months, *P* = 0.02) were significantly associated with decreased OS. Moreover, positive PD1 reaction in tumor cells (*P* = 0.03) (Fig. 3A) and TILs (*P* = 0.04) were significantly associated with prolonged OS (Table 3) (Fig. 3B).

**Table 3:** Univariate and multivariate analysis of factors affecting overall survival time (OS) in studied cases (*n* = 103)

Univariate analysis				Multivariate analysis		
Clinicopathological parameters	% censored cases	Median OS time (min–max) per months	Log-rank test	*Β*	*P*	Hazard ratio (95% CI)
Diabetes mellitus (DM)						
• No	(22.1%)	37.8 (30.3–45.4)	*χ*^2^ = 4.7			1
• Yes	(12.5%)	15.3 (7.1–23.4)	*P* = 0.03*	1.1	0.02*	2.8 (1.2–6.6)
IPI score						
• Low	(21.2%)	37.8 (28.1–47.5)	*χ*^2^ = 9.8			1
• Low-intermediate	(21.6%)	41.1 (27.1–55.1)	*P* = 0.02*	0.06	0.83	0.94 (0.53–1.7)
• High- intermediate	(28%)	28.7 (20–37.4)		0.17	0.59	1.2 (0.64–2.2)
• High	(0%)	13.3 (8.9–17.6)		1.2	0.004*	3.4 (1.5–7.7)
PD1						
In TILs						
• Negative	(16.1%)	24.5 (17.6–31.4)	*χ*^2^ = 4.2			1.4 (0.87–2.3)
• Positive	(23.6%)	40.5 (31.4–49.7)	*P* = 0.04*	0.34	0.17	1
In tumor cells						
• Negative	(19.4%)	33.7 (27.7–39.6)	*χ*^2^ = 4.6			2.6 (0.59–11.3)
• Positive	(60%)	81.6 (27.5–135.7)	*P* = 0.03*	0.95	0.21	1
LAG3						
In TILs						
• Negative	(22.2%)	44.5 (27.9–61.1)	*χ*^2^ = 1.4	–	–	–
• Positive	(21.2%)	35.2 (26.6–43.8)	*P* = 0.23			
In tumor cells						
• Negative	(21.1%)	36.6 (29.4–43.9)	*χ*^2^ = 0.51	–	–	–
• Positive	(25%)	20.5 (11.4–43)	*P* = 0.47			
CTLA4						
In TILs						
• Negative	(12.5%)	34.9 (9.2–60.5)	*χ*^2^ = 0.01	–	–	–
• Positive	(22.1%)	36.7 (28.8–44.7)	*P* = 0.93			
In tumor cells						
• Negative	(22.8%)	37.8 (29.4–46.3)	*χ*^2^ = 0.81	–	–	–
• Positive	(16.7%)	28.9 (20–37.9)	*P* = 0.37			

*Significant P ≤ 0.05

*c*^*2*^ chi-square test, *β* beta *CI* confidence interval, *r* reference group

While not statistically proven, LAG3 expression in tumor cells and TILs was linked to shorter median OS (20.5 and 35.2 months) compared to cases negative for LAG3 (36.6 and 44.5 months), however it was statistically insignificant (*P* value 0.47 and 0.23 respectively). CTLA4 expression was not significantly associated with OS.

Multivariate Cox regression identified diabetes mellitus (HR = 2.8, 95% CI 1.2–6.6, *P* = 0.02) and high IPI score (HR = 3.4, 95% CI 1.5–7.7, *P* = 0.004) as independent predictors of lower OS in DLBCL patients (Table 3).

Shorter disease-free survival (DFS) was significantly associated with involvement of multiple (more than one) extra-nodal sites (*P* = 0.045), positive bone marrow aspirate (*P* = 0.004), and positive bone marrow biopsy (*P* = 0.02). LAG3 expression in tumor cells was also significantly associated with shorter DFS (*P* = 0.04) (Fig. 3C). Although CTLA4 expression in tumor cells and TILs was associated with lower DFS times (23.5 and 34.2 months, respectively), these associations were not statistically significant (Table 4).

**Table 4:** Univariate and multivariate analysis of factors affecting disease-free survival time (DFS) in the studied cases (*n* = 103)

Univariate analysis				Multivariate analysis		
Clinicopathological parameters	% censored cases	Median OS time (min–max) per months	Log-rank test	*β*	*P*	Hazard ratio (95% CI)
Extra nodal sites						
• No	(53.2%)	38.7 (30.3–47.2)	*χ*^2^ = 5.8			1
• 1 (one site)	(27.3%)	23.3 (15.2–31.3)	*P* = 0.045*	0.45	0.22	(0.76–3.2)
• 2 (more than 1 site)	(50%)	12 (10.4–13.6)		0.68	0.38	1.9 (0.43–9.2)
Bone marrow aspiration						
• No	(50.5%)	37.3 (29.8–44.7)	*χ*^2^ = 8.2			1
• Yes	(25%)	16.9 (10.1–23.8)	*P* = 0.004*	9.1	0.004*	2.6 (1.7–5.1)
Bone marrow biopsy						
• No	(49.4%)	37 (29.6–44.4)	*χ*^2^ = 5.8			1
• Yes	(35.7%)	18.1 (11.1–25.2)	*P* = 0.02*	8.4	0.006*	2.2 (1.1–7.3)
LAG3 in tumor cells						
• Negative	(46.5%)	35.1 (28.2–41.8)	*χ*^2^ = 5.9			1
• Positive	(75%)	9.3 (6.7–12.1)	*P* = 0.04*	1.3	0.21	3.6 (0.48–27.9)
PD1						
In TILs						
• Negative	(51.6%)	25.6 (19.7–31.5)	*χ*^2^ = 0.77	–	–	–
• Positive	(45.8%)	36.8 (28.7–44.9)	*P* = 0.38			
In tumor cells						
• Negative	(49%)	31 (26.4–35.6)	*χ*^2^ = 0.34	–	–	–
• Positive	(20%)	32.7 (19.9–71.7)	*P* = 0.56			
LAG3 in TILs						
• Negative	(44.4%)	34.3 (23.8–44.9)	*χ*^2^ = 0.14	–	–	–
• Positive	(48.2%)	35.9 (27.8–44)	*P* = 0.71			
CTLA4						
In TILs						
• Negative	(75%)	48 (44.6–49.4)	*χ*^2^ = 2.2	–	–	–
• Positive	(45.3%)	34.2 (27.1–41.3)	*P* = 0.14			
In tumor cells						
• Negative	(49.4%)	36.9 (29–44.9)	*χ*^2^ = 1.7	–	–	–
• Positive	(41.7%)	23.5 (18–29.1)	*P* = 0.19			

*c*^*2*^ chi-square test, *β* beta *CI* confidence interval, *r* reference group

*Significant *P* ≤ 0.05

Multivariate Cox regression analysis identified positive bone marrow aspiration (HR = 2.6, 95% CI 1.7–5.1, *P* = 0.004) and positive bone marrow biopsy (HR = 2.2, 95% CI 1.1–7.3, *P* = 0.006) as independent prognostic predictors for shorter DFS in DLBCL patients (Table 4).

## Discussion

Inhibitory receptors PD1, LAG3, and CTLA4, expressed by immune cells within the TME, can suppress anti-tumor immunity and foster cancer cell growth [19]. This study assessed the IHC expression of these markers in DLBCL tumor cells and TILs, correlating their expression with clinicopathological parameters.

In our study, PD1 and LAG3 expression was predominantly found on TILs (71% and 85%, respectively) compared to tumor cells (4.9% and 3.9%). This aligns with findings by Chen et al. (2019) in DLBCL, who reported low PD1 and LAG3 expression on tumor cells (8.3% and 7.5%, respectively) but high expression on TILs (77% and 84.7%, respectively) (15).

PD1 expression demonstrated insignificant association with the majority of clinical and laboratory parameters examined. However, a statistically significant association was observed with HCV positivity, aligning with findings by Huang et al. (2021) [20], who documented elevated PD1 expression on CD4 + and CD8 + T-cells in patients with HCV infection, this was explained by that One of the major mechanisms driving the persistence of HCV infection is T-cell exhaustion, which results in weak antigen-specific T-cell responses. Persistent HCV antigen stimulation leads to exhaustion of virus-specific T-cells, characterized by changes in the expression of multiple co-signaling molecules, including PD1, which limit proliferative capacity, impair cytokine secretion, and reduce the elimination of HCV [20].

In TILs, LAG3 expression is significantly associated with the IPI score, with 96% of cases with high-intermediate risk exhibiting LAG3 positivity. No significant association was found between LAG3 and other parameters. Ma et al. (2023) reported a link between PD1 and LAG3 expression in DLBCL TILs and bone marrow involvement, suggesting that combined PD1 and LAG3 blockade may restore CD8 + T cell activity and facilitate personalized cellular immunotherapy for DLBCL, that study hypothesized that blockage of LAG-3 and PD-1 may improve PD-1 resistance for patients in whom PD-1 treatment is ineffective and tumors are drug-resistant [21]. Anti-LAG-3 blockade also showed strong activity in cultured T-cells directed against DLBCL cell lines as suggested by Chen et al., 2019 [15].

Lee et al., (2023) suggests that the combination of anti-PD-1/PD-L1 and anti-LAG-3 inhibitors in the immunotherapy of DLBCL patients can have a synergistic effect and broaden the range of the TMEs covered by immunotherapy, improving the immunotherapy efficacy and outcome in DLBCL patients [22].

CTLA4 expression in TILs was found in 92.2% of cases and was significantly associated with disease stage. Notably, all stage IV cases expressed CTLA4. CTLA4 positivity was associated with advanced stages (III/IV), consistent with findings reported by Rifani et al. (2025) [23].

While univariate analysis in our research revealed a significant association between PD1 + TILs and prolonged OS, aligning with some prior studies [24–27], other reports have linked high PD1 expression to inferior OS [21, 28, 29]. Conversely, Chen et al. (2019) found no association between PD1 and LAG3 expression on TILs and patient outcome [15]. These conflicting results may stem from variations in sample size and assessment methods.

While not statistically significant, our findings suggest a potential association between LAG3 expression in TILs and reduced OS time, aligning with previous studies demonstrating that elevated LAG3 expression is associated with unfavorable prognoses (Tobin et al., 2021 [30], Keane et al., 2021 [31], and Ma et al., 2023 [21].

Both univariate and multivariate analyses indicated that diabetes mellitus was significantly associated with lower OS, and was an independent prognostic predictor. This finding aligns with Han et al. (2022) [32].

Univariate analysis revealed that LAG3 expression in tumor cells and involvement of multiple extra-nodal sites was significantly associated with lower DFS. These findings align with prior reports by Keane et al. (2021) [31], who linked high LAG3 levels to inferior DFS, and Bobillo et al. (2021) [33], who associated multiple extra-nodal sites with decreased DFS.

Our study demonstrated that positive bone marrow aspirate (BMA) and bone marrow biopsy (BMB) were significantly associated with lower DFS. Multivariate Cox regression analysis confirmed that bone marrow aspirate and biopsy are independent prognostic predictors for lower DFS, a finding supported by Alonso-Álvarez et al. (2020) [34].

There were some limitations in our study: (1) it was a single-center study; thus, there could be some selective biases; (2) our study was done using IHC method for evaluation of PD1, LAG3, and CTLA4 expression, further studies using both gene analysis and IHC methods are needed; (3) this study did not investigate the precise mechanism by which these immune checkpoint molecules contributes to the pathophysiology of DLBCL, which is needed to be further investigated. (4) The small number of cases (*n* = 5) with positive PD1 reaction in tumor cells may limit the strength of the association with overall survival.

## Conclusion

PD1 and LAG3 are expressed mainly in TILs in DLBCL. PD1 expression (in TILs and tumor cells) is associated with prolonged OS, while LAG3 expression (in tumor cells) is associated with shorter DFS and its expression in TILs tended towards shorter OS. CTLA4 expression is associated with advanced disease stage but not associated with OS. These findings may suggest that immune checkpoint inhibitors targeting LAG3 may offer therapeutic potential in DLBCL by enhancing the antitumor immune response. Additional research is needed to assess the effectiveness of inhibition of these checkpoint molecules in combination with existing treatment modalities.

## Data Availability

The datasets used during the current study are available from the corresponding author on reasonable request.

## Abbreviations

BMA: Bone marrow aspirate
BMB: Bone marrow biopsy
CI: Confidence interval
CTLA4: Cytotoxic T-lymphocyte-associated protein 4
DFS: Disease-free survival
DLBCL: Diffuse large B cell lymphoma
H&E: Hematoxylin and eosin
HR: Hazard ratio
IC: Immune checkpoint
IHC: Immunohistochemical
IPI: International Prognostic Index
Lag3: Lymphocyte-activation gene-3
MHC: Major histocompatibility complex
NCI: National Cancer Institute
NHL: Non-Hodgkin lymphoma
NOS: Not otherwise specified
OCMU: Oncology Center of Mansoura University
OS: Overall survival
PD1: Programmed cell death protein 1
TILs: Tumor-infiltrating lymphocytes
TMA: Tissue microarray
TME: Tumor microenvironment
Tregs: T regulatory cells
